# TRIM25 dictates selective miRNA loading into extracellular vesicles during inflammation

**DOI:** 10.1038/s41598-023-50336-5

**Published:** 2023-12-22

**Authors:** Kayla E. King, Priyanka Ghosh, Ann L. Wozniak

**Affiliations:** 1 Department of Internal Medicine, University of Kansas Medical Center, Mailstop 1018, Kansas City, KS 66160 USA; 2 Liver Center, University of Kansas Medical Center, Kansas City, KS 66160 USA

**Keywords:** Multivesicular bodies, ESCRT, Cell biology

## Abstract

Extracellular vesicles (EVs) such as exosomes are loaded with specific biomolecules in order to perform cell-to-cell communication. Understanding the mechanism of selective cargo loading is important to better understand the physiological and pathological function of EVs. Here we describe a novel target of the E3 ligase TRIM25 and show that inflammation-mediated EV loading of the RNA binding protein FMR1 and its associated microRNA, miR-155, is promoted by TRIM25-mediated K63-ubiquitination of FMR1. This ubiquitination promotes an interaction between FMR1 and the EV loading machinery via the cleavage of the trafficking adaptor protein RILP. These interactions are lost when TRIM25 is knocked down. Loss of TRIM25 also prevents the loading of both FMR1 and miR-155. These findings suggest that inflammation-mediated loading of FMR1 and its associated microRNAs into the EV are dependent on K63-ubiquitination by TRIM25 and provide novel insights and tools to manipulate EV biogenesis for therapeutic benefit.

## Introduction

Extracellular vesicles (EVs), including exosomes, contain a specific set of lipids, proteins, and nucleic acids that, when taken up by a recipient cell, modulate immunity and inflammation by conferring pathogenic or therapeutic effects^1^. EV cargo is not simply a reflection of total cellular content, indicating the presence of tightly controlled and highly regulated EV sorting mechanisms. Several potential mechanisms for EV cargo sorting have been described and can be broadly classified as ESCRT-dependent or ESCRT-independent^2^. The Endosomal Sorting Complex Required for Transport (ESCRT) consists of four distinct complexes that regulate the biogenesis of the multivesicular body (MVB) and is directly implicated in the formation of intralumenal vesicles (ILVs) as well as the sorting of cargo into them. EV cargo sorting involves a ubiquitinated protein being recognized by the ESCRT-0 complex which is composed of Hrs and STAM1/2. It has been suggested that the ESCRT complex typically recognizes K63-ubiquitinated proteins^3^.

Ubiquitination is performed by a series of proteins, the last being an E3 ligase. E3 ligases catalyze the conjugation of ubiquitin to a target protein. Ubiquitination is divided into mono-ubiquitination or poly-ubiquitination whereby a chain of ubiquitin can be attached via 7 lysine residues (K6, K11, K27, K29, K33, K48, and K63)^4^. Ubiquitin-K48 and -K63 are the best-characterized residues involved in ubiquitination. The different types of ubiquitination lead to various fates of substrate proteins. K48-linked ubiquitination labels proteins for proteasome-mediated recognition and degradation while K63-linked ubiquitination is involved in signaling complexes^5^. The tripartite motif (TRIM) family of E3 ligases regulate several cellular processes including cell growth, differentiation, cancer development, and innate immune response^6,7^. TRIM25 is well known as an interferon-inducible E3 ligase whereby it initiates intracellular antiviral signaling by inducing the K63-linked ubiquitination of RIG-I^8^. It has also been shown to ubiquitinate other targets including RNA-binding proteins^6,9,10^.

RNA binding proteins (RBPs) play a key role in regulating the post-transcriptional processing and transport of RNA molecules, particularly during inflammation where they play essential roles in the regulation of immune cell responses via stabilization and/or degradation of mRNA transcripts^11,12^. More recently, RBPs have been directly implicated in EV miRNA sorting^13,14^. We previously showed that inflammasome activation results in the exosomal loading of the RBP fragile X mental retardation 1 (FMR1) and its associated microRNA, miR-155^15^. The mechanism by which this occurs is dependent on cleavage of RILP (Rab interacting lysosomal protein), a Rab7 effector that directs trafficking through the endo-lysosomal pathway via direct interactions with dynein motor complexes^16^. RILP undergoes caspase-1 dependent cleavage during inflammation, resulting in the altered trafficking of intracellular vesicles including the multivesicular body, the site of exosome biogenesis^17^. Interestingly, the cleavage status of RILP directly influences which cargo are put into the exosome. Overexpression of cleaved RILP (cRILP) alone results in the loading of FMR1 and miR-155, mimicking the effects on exosome loading seen after full inflammasome activation^15^. When a non-cleavable form of RILP (ncRILP) is expressed, the inflammasome-dependent loading of FMR1 is prevented. This suggests the existence of distinct RILP-mediated sorting mechanisms.

We sought to determine the mechanism of inflammasome-mediated loading of FMR1 and to investigate the role of RILP in this process. We found that inflammation promotes the K63-ubiquitination of FMR1, and RILP cleavage is sufficient to promote this ubiquitination. We discovered that the E3 ligase TRIM25 is responsible for not only ubiquitinating FMR1 but also inducing its loading, along with its associated microRNAs, into the EV by promoting an interaction with the ESCRT-0 complex.

## Results

### TRIM25 mediates FMR1 K63-ubiquitination

**Figure 1:**
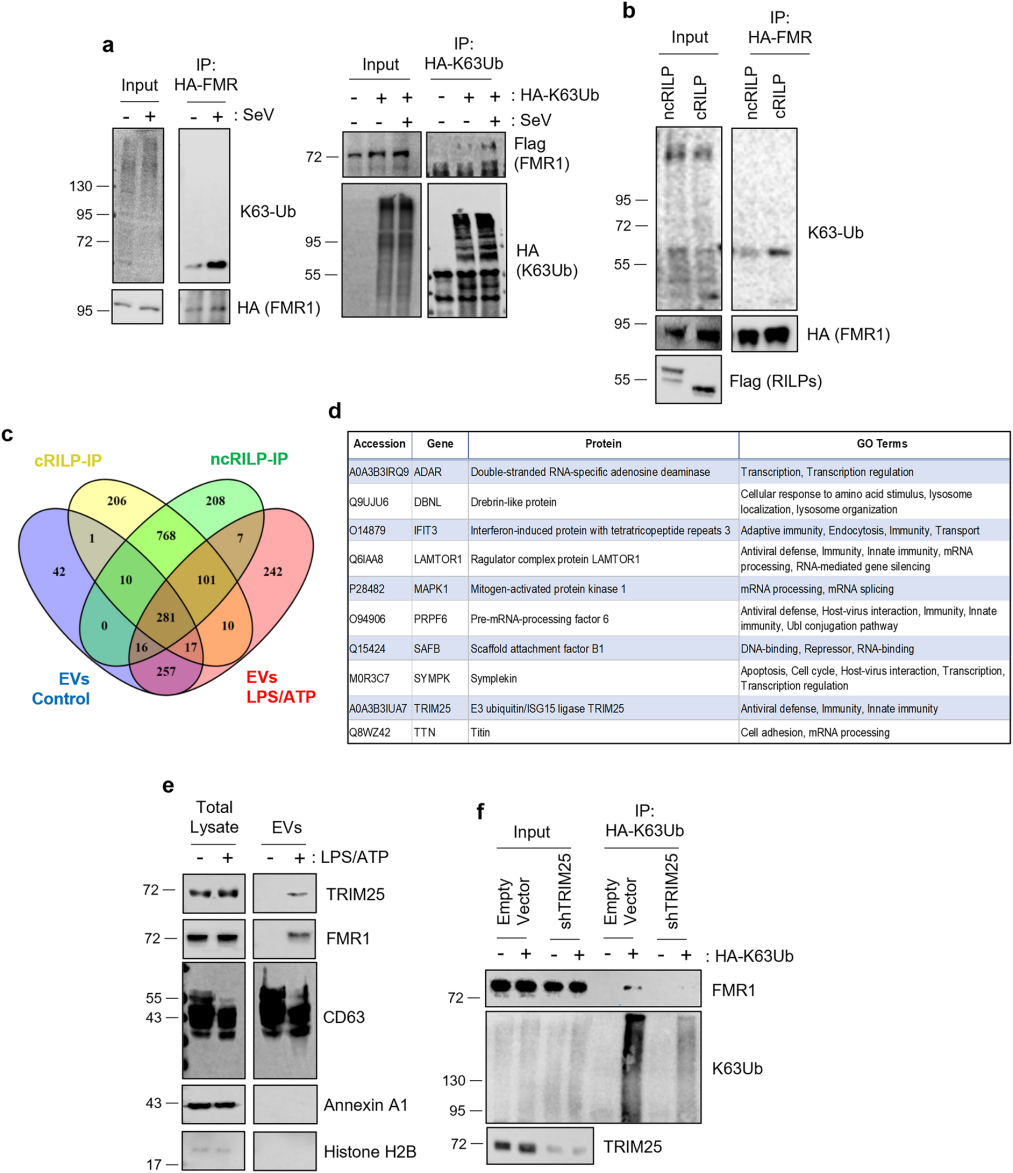
TRIM25 Mediates FMR1 K63-ubiquitination. (**A**) Left panel shows a western blot analysis of HA-FMR1 immunoprecipitated from HeLa cells revealing that FMR1 is K63-Ub, particularly during inflammatory conditions. The right panel confirms FMR1 ubiquitination via a reversal IP. (**B**) Expression of cleaved RILP (cRILP) alone promotes the K63-Ub of FMR1 in HeLa. (**C**) Venn diagram of mass spectrometry analysis from proteins that immunoprecipitated with cRILP or ncRILP as well as EVs isolated from control, untreated THP-1 cells or inflammatory EVs isolated from THP-1 cells treated with LPS/ATP. (**D**) Cross-referencing the proteins that directly associate with cRILP and are loaded into inflammatory EVs yields a list of 10 proteins. One protein, TRIM25, is an E3 ligase. (**E**) In THP-1 cells, both TRIM25 and FMR1 are enriched it the EVs induced by inflammasome activation. (**F**) shRNA knockdown of TRIM25 in THP-1 cells blocks the K63 ubiquitination of FMR1. n = 3–5.

In previous work, we showed that the efficient loading of the RNA binding protein FMR1 into the extracellular vesicle (EV) during inflammation requires both inflammasome activation and association with ESCRT complexes^15^. ESCRT-dependent EV cargo sorting involves a K63-ubiquitinated protein being recognized by the ESCRT-0 complex. We therefore examined FMR1 ubiquitination in relation to inflammasome status. HeLa cells were transfected with HA-FMR and infected with Sendai Virus to activate the inflammasome. Lysates were collected and FMR was immunoprecipitated with anti-HA magnetic beads. Western blot analysis of the immunoprecipitates for K63 ubiquitination showed that not only is FMR K63 ubiquitinated, but its ubiquitination is upregulated after inflammasome activation (Fig. 1A, left panel). We confirmed FMR-1 ubiquitination via a reversal IP, HA-K63Ub pulldown and western blot for FLAG-FMR1 (Fig. 1A, right panel).

Inflammasome activation results in caspase-1 mediated cleavage of RILP and this event is sufficient to promote the loading of FMR1 into the EV/exosome^15,17^. Previous data also showed that the cleaved form of RILP (cRILP) promotes the interaction between FMR1 and components of the ESCRT complex^15^. Therefore, we next assessed the effect of RILP cleavage on FMR1 ubiquitination. HeLa cells were transfected with HA-FMR and either cRILP or a dominant-negative, non-cleavable RILP, ncRILP. Lysates were collected and FMR1 was immunoprecipitated as stated previously. The K63-ubiquitination of FMR1 increased with the expression of cRILP compared to the ncRILP condition (Fig. 1B). Altogether, this data shows that inflammasome activation and subsequent cleavage of RILP results in the K63-ubiquitination of FMR1.

Next, we determined which E3 ligase was responsible for ubiquitinating FMR1. For this set of experiments, we used THP-1 cells, a human monocytic cell line that expresses high levels of the NLRP3 inflammasome components and differentiated them with PMA to induce a macrophage phenotype^18^. To generate a list of E3 ligase candidates, we performed a systematic mass spectrometry analysis on a series of samples; cellular proteins that associate with cRILP or ncRILP as well as EVs isolated from untreated THP-1 cells or inflammatory EVs isolated from THP-1 cells treated with LPS/ATP. Because FMR is K63-ubiquitinated after an inflammatory stimulus, we reasoned that the E3 ligase would directly associate with cRILP and be loaded into inflammatory EVs while being excluded from non-inflammatory EVs. Cross-referencing proteins that were only associated with cRILP and only loaded into inflammatory EVs resulted in a list of 10 proteins. Only one was an E3 ligase: TRIM25 (Fig. 1C,D). To confirm the role of TRIM25 in the EV loading of FMR1, we first assessed its presence in the EV. THP-1 monocytes were differentiated into macrophages using PMA and treated with LPS/ATP to stimulate the inflammasome and thus RILP cleavage. TRIM25 was enriched in the EVs induced by inflammasome activation, as was FMR1 (Fig. 1E). We next assessed whether TRIM25 directly ubiquitinates FMR1. Immunoprecipitation of K63-Ub shows that FMR1 is K63-ubiquitinated (Fig. 1F). shRNA knockdown of TRIM25 blocked the K63 ubiquitination of FMR. Altogether, the data shows that TRIM25 mediates the K63Ub of FMR1.

### K63-ubiquitinated FMR1 undergoes ESCRT-dependent extracellular vesicle loading

**Figure 2:**
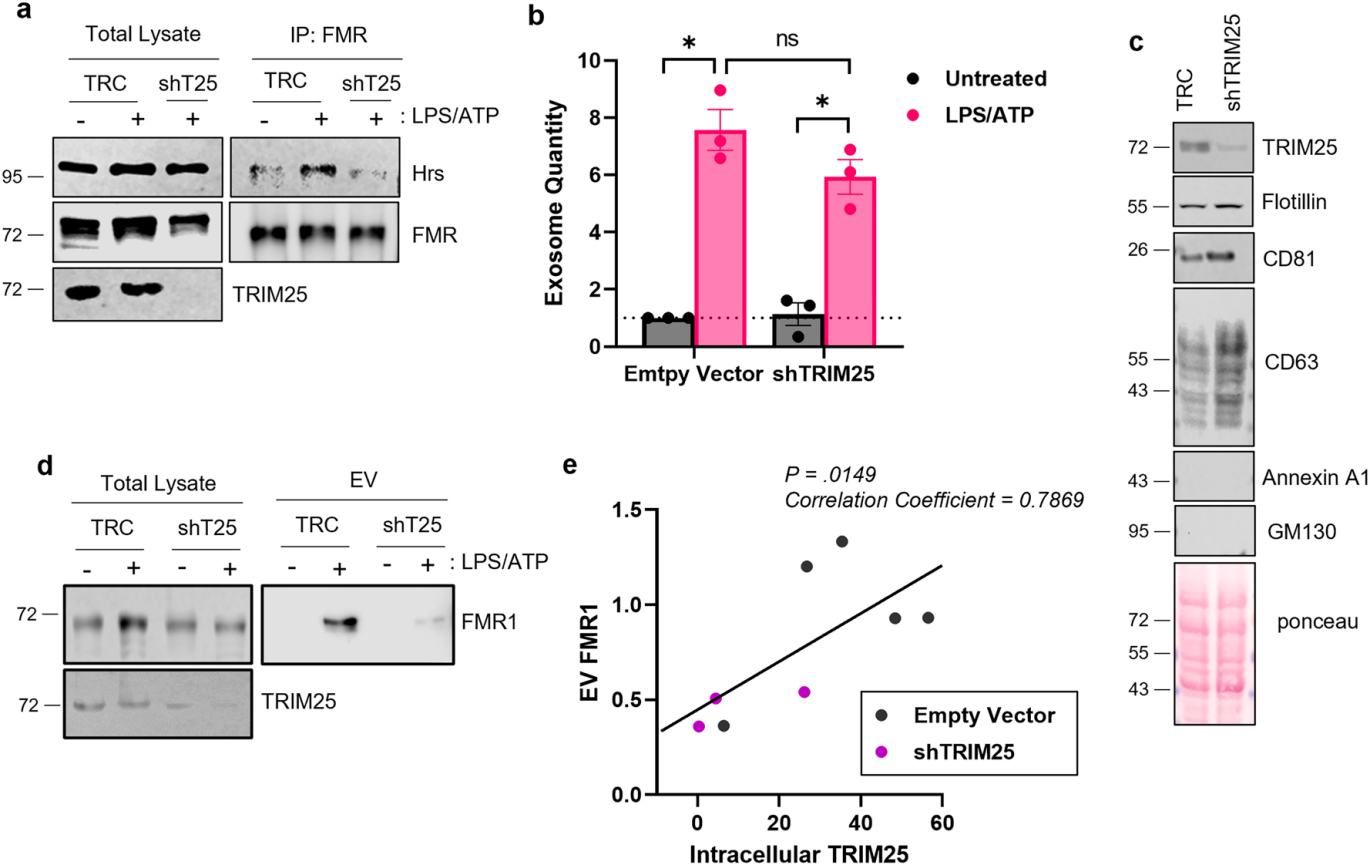
K63-Ubiquitinated FMR1 undergoes ESCRT-dependent EV loading. (**A**) Pulldown of FMR1 shows that it interacts with Hrs after inflammasome activation. This interaction is lost when TRIM25 is silenced (shT25). (**B-C**) Knockdown of TRIM25 does not alter the number of EVs secreted or the contents of known exosome markers. EV numbers were quantified via Bradford dye as performed by us previously^15^. (**D**) Silencing TRIM25 dramatically reduces the EV enrichment of FMR1 during inflammation. (**E**) Correlation between the amount of intracellular TRIM25 and the amount of EV-associated FMR1. The amount of intracellular TRIM25 is positively correlated with EV FMR1. THP-1 cells were used for this figure. Data are shown as mean ± SEM; *p ≤ 0.05 for n = 3–5.

The Endosomal Sorting Complex Required for Transport (ESCRT) consists of four distinct complexes. EV cargo sorting involves a K63-ubiquitinated protein being recognized by the Hrs component of the ESCRT-0 complex. Therefore, we next examined the effect of K63-ubiquitination on the ability of FMR1 to interact with Hrs. To assess this, we performed immunoprecipitation of FMR1 under control or TRIM25 knockdown conditions and probed for Hrs. Supporting previous data^15^, FMR1 interacts with Hrs after inflammasome activation with LPS/ATP (Fig. 2A). However, the interactions between FMR1 and Hrs are lost when TRIM25 is knocked down. This data supports that K63-ubiquitinated FMR1 interacts with ESCRT machinery.

Because knockdown of TRIM25 reduced the interaction between FMR1 and the ESCRT machinery, we next assessed whether TRIM25-mediated ubiquitination of FMR1 was necessary to promote the EV loading of FMR1. Knockdown of TRIM25 did not alter EV output as assessed by quantity (Fig. 2B) or EV content assessed by the presence of proteins known to be present in exosome-like vesicles (Fig. 2C). However, silencing TRIM25 dramatically reduced the EV enrichment of FMR1 (Fig. 2D). Due to variations in TRIM25 knockdown efficiency and FMR1 expression levels, we correlated the amount of intracellular TRIM25 to the amount of EV FMR1. The correlation shows that the amount of intracellular TRIM25 is positively correlated with EV FMR1 (Fig. 2E). Taken together, these data suggest that TRIM25 promotes FMR1 EV loading.

### FMR ubiquitination is required for FMR1-mediated miRNA loading

**Figure 3:**
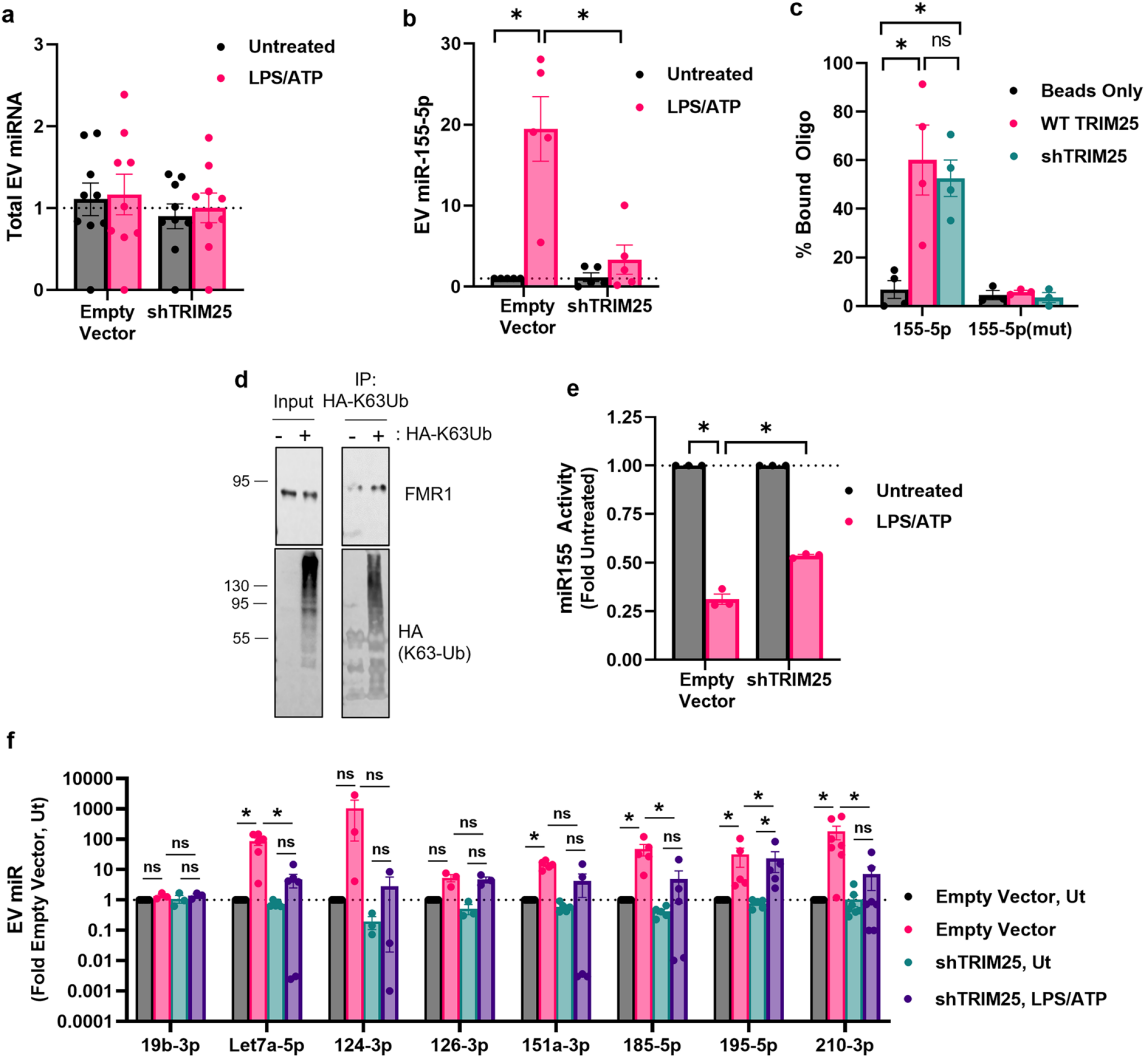
FMR Ubiquitination is Required for FMR1-mediated miRNA loading. (**A**) Total EV miRNA is not affected by TRIM25 knockdown in THP-1 cells. (**B**) Using THP-1 cells, miR-155 is enriched in inflammatory EVs. This effect is blocked when TRIM25 is silenced. (**C**) Analysis of FMR1-bound miR-155. FMR1 purified from both wild-type and sgTRIM25 CRISPR HeLa cells binds miR-155. FMR1 did not bind miR-155(mutant), a mutant engineered to eliminate the FMR1 binding motif. (**D**) FMR1 is K63Ub in HMC3 cells. (**E**) miR-155-5p is transferred and functional in a target cell. Conditioned media from an LPS/ATP-treated HMC3 macrophage reduces luciferase activity in a target expressing pmiRGlo-based luciferase reporter plasmid containing two miR-155-5p binding sites indicating the transfer of a functional miR-155-5p. Less functional miR-155-5p is transferred when target cells are treated with conditioned media from LPS/ATP-treated TRIM25 knockdown cells. (**F**) Using THP-1 cells, TRIM25 influences the EV loading of several miRNAs. Data are shown as mean ± SEM; *p ≤ 0.05 for n = 3–5.

We previously observed that the loading of miR-155-5p into the EV is regulated by FMR1^15^ and here we show that TRIM25 regulates the loading of FMR1 into the EV. Therefore, we next examined the role of TRIM25 in miR-155-5p EV loading. EV miRNA was purified from control and TRIM25 knockdown cells. We first confirmed that knockdown of TRIM25 does not alter the amount of miRNA secreted in the EVs (Fig. 3A). LPS/ATP treatment increased miR-155-5p in the EV. This effect was blocked when TRIM25 was silenced (Fig. 3B). To ensure that the effect of TRIM25 on the EV loading of miR-155-5p was not a result of decreased binding to FMR1, we examined the ability of FMR1 to bind miR-155-5p when TRIM25 is knocked down. We chose to use Sendai virus (SeV) infected Hela cells owing to their ease of transfection. HA-FMR was immunopurified from SeV infected wild-type or TRIM25 knockdown cells using HA-tagged magnetic beads. FMR1-bound beads were incubated with FAM-tagged miR-155 RNA. After 3 h, the beads were washed, bound miRNA was eluted from the beads, and FAM fluorescence in the eluate was determined by a plate reader. Analysis of the FMR1-bound miRNA shows that FMR1 purified from wild-type HeLa cells binds miR-155-5p. TRIM25 knockdown did not affect this binding (Fig. 3C). To assure that these findings were due to FMR1-miR-155-5p binding, we repeated the experiment using miR-155(mutant) that was engineered to eliminate the FMR1 binding motif^15^. FMR1 did not bind miR-155(mutant). These data support that miR-155-5p is loaded in an FMR1-dependent manner and suggest that miR-155-5p loading is dependent on TRIM25-mediated FMR1 ubiquitination.

In order to determine if the decreased EV loading of miR-155-5p after TRIM25 knockdown makes a functional difference in a receiving cell, we performed a conditioned media luciferase reporter assay. We used HMC3 macrophage cells for ease of transfection. We first confirmed FMR1 K63 ubiquitination in these cells. The cells were transfected with an HA-tagged K63Ub. Lysates were collected and K63Ub was immunoprecipitated with anti-HA magnetic beads. Western blot analysis of the immunoprecipitates for FMR1 showed that it is K63 ubiquitinated (Fig. 3D). Next, control or TRIM25 knockdown HMC3 cells were treated with LPS and ATP to stimulate the inflammasome. Cell culture supernatants were collected, and this conditioned media was used to treat target cells expressing a pmiRGlo-based luciferase reporter plasmid containing two miR-155-5p binding sites. When targets cells were treated with LPS/ATP-treated conditioned media, luciferase activity decreased indicating that miR-155-5p was being transferred and downregulating the target luciferase RNA (Fig. 3E). When target cells were treated with conditioned media from LPS/ATP treated TRIM25 knockdown cells, luciferase activity in the target cell was less affected. This indicates that EV miR-155-5p plays a functional role in receiving cells and can be altered by TRIM25 knockdown.

We then sought to determine if TRIM25 influences the EV loading of other cargos in addition to FMR1 and miR-155-5p. We collected EV miRNA from control or TRIM25 knockdown cells with or without LPS/ATP treatment. We found that TRIM25 influenced the loading of several microRNAs, including Let7a-5p, 185-5p. 195-5p, and 210-3p (Fig. 3F). However, not all microRNAs we screened were influenced by TRIM25. This suggests that TRIM25 regulates the loading of a broad yet specific subset of inflammatory EV microRNAs.

## Discussion

Extracellular vesicles (EVs) including exosomes contain a specific set of cellular content that can alter the phenotype of a target cell. Because they have been implicated in the progression of diseases, particularly those with an inflammatory component^1,19–21^, it is important to understand the mechanism by which cargo are selectively sorted into the EV, as these mechanisms may represent future clinical targets. In this study, we show that EV microRNA loading is influenced by posttranslational modifications of its RNA binding protein and reveal a novel mechanism by which the E3 ligase TRIM25 regulates EV loading during inflammation. The data show that TRIM25 is responsible for not only K63-ubiquitinating the RNA binding protein (RBP) FMR1, but also inducing its loading into the EV by promoting an interaction with the Hrs component of the ESCRT-0 complex. Interactions between FMR1 and Hrs are lost when TRIM25 is silenced thus preventing the EV enrichment of FMR1 as well as its associated miRNA without affecting the ability of FMR1 to bind miR-155-5p.

These data highlight a previously unknown target of TRIM25, an E3 ligase most known for its role in RIG-I signaling during the antiviral response^6,7^. While no evidence exists linking TRIM25 to EV biogenesis, it has been shown to ubiquitinate other targets including RNA-binding proteins. This ubiquitination is often required for RBP-RNA binding. For example, TRIM25 modulates the ability of ZAP to bind its target RNA^10^. In addition, TRIM25 is required for the binding of Lin28a to the precursor of let-7 (pre-let-7)^9^. However, our data reveals that TRIM25 is not necessary or required for FMR1 to bind its target miRNA, miR-155. It is, however, required for the FMR1-miR-155 complex to load into the EV. This suggests that the ubiquitination of FMR1 functions more as a signaling modification to be recognized by the ESCRT complex. This is true for other E3 ligases including Nedd4 which binds to and ubiquitinates proteins for their eventual recruitment to the MVB^22^. In further support, other EV-targeting protein modifications for RBPs have been seen including SUMOylation and ISGylation^23,24^.

We noted that total cellular K63-ubiquitination decreased when TRIM25 was silenced. It is therefore possible that the effect of TRIM25 on FMR1 ubiquitination and eventual EV loading is not a direct interaction but instead a result of TRIM25 acting on another protein which would in turn initiate FMR1 ubiquitination. This is seen with several of the Nedd4 E3 ligases which can bind to and ubiquitinate target proteins either by themselves or through an adaptor protein^22^. However, our data does not suggest the requirement of an adaptor protein; TRIM25 is enriched in the EV yet no adaptor protein is present, cRILP alone promotes TRIM25-mediated FMR1 ubiquitination, and FMR1 binds miR-155 even in the absence of TRIM25. In any case, the data clearly show a direct dependence between the EV loading of FMR1 and the presence of TRIM25.

TRIM25 knockdown decreases the amount of functional miR-155-5p transferred to a target cell. Additionally, TRIM25 is not limited to influencing the loading of one microRNA. The data show that TRIM25 regulates EV loading of several microRNAs. It is likely that it is affecting the loading of these microRNAs by ubiquitinating their unique RBP partners, following the precedent set here by FMR1 and miR-155-5p. This suggests that TRIM25 may be important for regulating the loading and EV transfer of a specific subset of RBPs and microRNAs during inflammation.

TRIM25 may be a viable therapeutic target for treating inflammatory conditions. There are many ongoing efforts to target E3 ligases, however, inhibitor development remains challenging^25^. It may be easier to instead target RNA binding proteins implicated in inflammatory disease. Here, we show that preventing the EV loading of FMR1 by knocking down TRIM25 reduces EV miR-155-5p, which results in less functional miR-155-5p in a target cell. miR-155-5p is known to be involved in inflammatory conditions^26^, so the ability to prevent its propagation may be beneficial in treating inflammatory disease. The same may be true for other targets of TRIM25 and is worth further investigation.

An increase in EV secretion is well known to occur during inflammation. Data presented here show that inflammation promotes the ubiquitination of FMR and does so by promoting an association between FMR1, TRIM25, and Hrs via the cleavage of RILP. In support, we found that the expression of cleaved RILP (cRILP) alone is sufficient to promote these interactions. This is further supported by the fact that when a dominant negative, non-cleavable RILP, ncRILP, was expressed, TRIM25 failed to ubiquitinate FMR1. Altogether, the results suggest that inflammation-mediated EV loading of FMR1 and its associated microRNAs are dependent on cRILP-mediated recruitment of TRIM25 and eventual recognition by the EV biogenesis machinery. This study provides insight into the mechanism behind inflammation-mediated selective EV loading and enhances our general understanding of EV cargo loading.

## Methods

### General materials and antibodies

General materials were purchased from Sigma-Aldrich or VWR. DMEM, FBS, RPMI, Opti-MEM, and Lipofectamine 3000 were purchased from Thermo Fisher Scientific. LPS was from Enzo (ALX-581-013-L002). Protease inhibitor cocktail (P8340; Sigma-Aldrich) was used at 1:100 dilution. Antibodies used included HA (Abcam, ab18181), TRIM25 (Novus, NBP2-61814), Lys63-specific ubiquitin (05-1308), TSG101 (EPR7130(B)), and Flag M2 (Sigma, F1804) were purchased from Sigma. Histone H2B (2722), Flotillin-1 (D2V7J), Hrs (D7T5N), GM130 (D6B1), and FMRP (4317S) were from Cell Signaling Technology. Horseradish peroxidase-conjugated secondary antibodies were from Thermo Scientific (Rockford, IL).

### Plasmids

pTRE2-Bla-cRILP and pTRE2-Bla-ncRILP have been described^27^. pFRT-TODest-FLAGHAhFMRPiso1 (Addgene plasmid 48690) was a gift from Thomas Tuschl^28^. HA-FMR1 was generated by excising the gene from pFRT and inserting into pLVX-IRES-mCherry followed by site-directed mutagenesis (NEB) to remove the Flag tag. All sequences were confirmed by DNA sequencing analysis. The pLKO-based plasmids to express shRNAs targeting TRIM25 (TRCN0000272649) and TRC1 empty vector control (SHC001) were obtained from Sigma-Aldrich. pRK5-HA-Ubiquitin-K63 was a gift from Ted Dawson (Addgene plasmid # 17606)^29^. 155_KRAS in pmiRGlo was a gift from Heidi Schwarzenbach (Addgene plasmid # 78132)^30^.

### Cells and culture conditions

HeLa cells (CRM-CCL-2) were purchased from American Type Culture Collection and cultured in DMEM containing 4.5 g/l glucose, L-glutamine, and sodium pyruvate, 10% FBS, and 1% nonessential amino acids. THP-1 human monocyte cells were purchased from ATCC (TIB-202) and cultured in RPMI 1640 containing 10% FBS. All cells were maintained at 37 °C/5% CO_2_. Transfections were performed using Lipofectamine 3000 according to manufacturer’s instructions. When indicated, SeV (Cantell strain, Charles River) was used at 400 hemagglutination units/ml for 24 h.

### Lentivirus production and transduction

For lentivirus production, 293FT cells were plated in 10-cm^2^ dishes and transfected with 1.95 µg psPax2, 650 ng pMDG.2, and 2.6 µg of either the pLKO-based shRNAs or the pLVX-IRES-mCherry-based plasmids using 15.6 µl of X-tremeGENE HP transfection reagent (Roche). The next day, the medium was replaced with antibiotic-free complete medium (DMEM supplemented with 10% FBS, 1 × MEM nonessential amino acids, 6 mM l-glutamine, and 1 mM sodium pyruvate). Supernatants were collected daily for the next 72 h, centrifuged at 3000 × *g* to remove dead cells, aliquoted and frozen at − 80 °C. To transduce THP-1 cells, the cells were plated at a concentration of 0.5 × 10^6^ cells per ml in 150-mm^2^ dishes. Lentivirus (10 ml) was incubated at room temperature with polybrene (8 µg/ml) for 15 min, then the virus was added to THP-1 cells and incubated overnight at 37 °C/5% CO_2_. The next day, the cells were differentiated into macrophage-like cells using PMA (100 ng/ml) for 4 h. The cells were washed, and the medium was replaced. After a 48-h rest period, the cells were washed in PBS and the medium was replaced with EV-free complete medium. When indicated, the cells were primed with 1 µg/ml LPS (serotype O55:B5, Enzo) for 3 h and then stimulated with 5 mM ATP for an additional 60 min to activate the inflammasome.

### Extracellular vesicle isolation and analysis

Cells (0.5 × 10^6^ cells per ml in 150-mm^2^ dishes) were cultured in 15 ml complete medium supplemented with 10% FBS (depleted of bovine EVs by overnight centrifugation at 100,000xg). EVs were isolated from cell culture supernatants via a series of differential ultracentrifugation steps as described in^31^. The final EV pellets were resuspended in PBS for further analysis. For total EV protein measurement, ≤ 5 µl of the resuspended EV pellet was assessed by standard Bradford dye assay^15^. When used for Western blotting, equal protein amounts were used.

### Immunoprecipitation

Whole-cell lysates were prepared from cells lysed in 1 × RIPA buffer supplemented with protease inhibitors by clarification at 20,000 × *g* for 15 min. HA-FMR1 was immunoprecipitated from approximately 500 µg total cell lysate by incubation with 25 µl anti-HA magnetic beads (Fisher, PI88837) overnight at 4 °C. The beads were washed five times with RIPA lysis buffer after which time they were incubated with 25 µl 2 × SDS sample buffer, heated at 95 °C for 5 min, and analyzed by SDS-PAGE.

### Western blotting

Whole-cell lysates were prepared from cells lysed in 1 × RIPA buffer supplemented with protease inhibitors. Cell lysates were separated using SDS-PAGE and transferred to polyvinylidene difluoride membranes (Millipore). Membranes were blocked with 5% milk and 0.1% Tween-20 in TBS for 1 h at room temperature. Membranes were incubated with the appropriate primary antibodies overnight at 4 °C. Finally, membranes were incubated with HRP-conjugated secondary antibodies for 1 h at room temperature and detected using the Pierce ECL Western blotting substrate kit (Thermo Fisher Scientific) and the Odyssey Fc, Dual-Mode Imaging system (Li-COR). Uncropped blots were provided (Supplementary Fig. 1).

### Mass spectrometry–cell preparation

THP-1 cells (six 150 cm^2^ culture dishes with 1 × 10^7^ cells per dish) were treated with PMA as stated above then primed with 1 µg/ml LPS (serotype O55:B5, Enzo) for 3 h and then stimulated with 5 mM ATP for an additional 60 min to activate the inflammasome. EVs were purified from culture supernatants and the final EV pellet was resuspended in 100 µl RIPA lysis buffer and used to perform mass spectrometry. For the immunoprecipitation samples, THP-1 cells (two 150cm^2^ culture dishes with 1 × 10^7^ cells per dish) were transduced with cRILP-Flag or ncRILP-Flag lentivirus followed by PMA treatment as stated above. Whole-cell lysates were prepared by adding 1.0 ml RIPA buffer supplemented with protease inhibitors followed by clarification at 20,000 × *g* for 15 min. The RILPs were immunoprecipitated from approximately 2000 µg total cell lysate by incubation with 50 µl anti-Flag magnetic beads (Sigma, M8823) overnight at 4 °C. The beads were washed five times with RIPA lysis buffer, followed by 3 washes in PBS. They were then resuspended in 100 µl RIPA buffer and sent for mass spectrometry.

### Proteolysis

100 μg of protein per condition were lyophilized and resuspended in 100 ul of 100 mM TEAB. Following reduction and alkylation, the proteins were acetone precipitated by the addition six volumes of chilled acetone and kept overnight at − 20 °C. After centrifugation the protein pellets were resuspended in 100 μl of 100 mM triethylammonium bicarbonate buffer (TEAB). After centrifugation, the protein pellets were resuspended in 100 ul of 50 mM TEAB and 2.5 ug of trypsin (Promega) were added per 100 μg of protein and incubated at 37 °C overnight. Trypsin digestion was stopped by the addition of 10% formic acid to a pH 2.0. Finally, all samples were lyophilized, and resuspended in 50 ul of 0.1% formic acid and an aliquot was injected into the HPLC coupled with the Orbitrap Fusion Lumos spectrometer.

### Mass spectrometric analysis

Peptide extracts were analyzed using a UHPLC system (nLC 1200, ThermoFisher Scientific) connected on line to an Orbitrap Fusion Lumos mass spectrometer (ThermoFisher Scientific). In brief, all samples were dissolved to a final volume of 450 µl of 0.1% formic acid and 10 μl was direct loaded on a C18 reverse phase peptide trap column (Acclaim PepMap 75 um × 2 cm TheremoFisher Scientific). Following a wash with 0.1% formic acid for 15 min at 0.5 µl/min, the flow of the peptide trap was diverted to the resolving column (Acclaim PepMap 75 um × 50 cm) and the peptides were eluted with a 5–80% acetonitrile gradient in 110 min at a flow rate of 300 nl/min.

All spectra were acquired using an Orbitrap Fusion Lumos mass spectrometer (ThermoScientific), controlled by Xcalibur 2.0 software (Thermo Scientific) and operated in data-dependent acquisition mode. FT MS1 spectra were collected at a resolution of 120,000, with an automatic gain control (AGC) target of 200,000 and a maximum injection time of 50 ms. Precursors were selected with an intensity threshold of 5000, according to charge state (to include charge states 2–7) and with monoisotopic precursor selection. Previously interrogated precursors were excluded using a dynamic window (60 s ± 10 ppm). An electrospray voltage of 2.7 kV was used, with the ion transfer temperature set to 275  °C. The mass spectrometer was controlled by the software to perform a 3 s cycle of mass analysis. The cycle consisted of one parent ion scan on the Orbitrap at 120,000 resolution, followed by MSMS scans on the Orbitrap analyzer at 40,000 resolution, top speed. A dynamic exclusion of one repeat scans of the same ion and 120 s exclusion duration was defined. Peptide fragmentation was done by HCD, the normalized collision energy was set to 35%.

### Data analysis and protein identification

For data analysis all MSMS scans were searched using Protein Discoverer v.2.4 running Sequest HT and a human database downloaded from the Uniprot data repository (uniprot-human_UP000005640.fasta) Full trypsin specificity was defined with a maximum of three missed cleavages and a precursor mass tolerance of 10 ppm and 0.02 Da for fragment tolerance. Carboxymethylation of Cys was used as a fixed modification. Met oxidation; deamidation of Asn and Gln; were used as variable modifications. A maximum of three equal modifications and four dynamic modifications was defined. Validation was based on the q-value obtained from Percolator.

### miRNA isolation, qPCR, and quantitation of EV miRNA enrichment

For miRNA analysis, the EV pellet was lysed in 1 ml QIAzol, and the miRNA was purified using the miRNeasy kit from Qiagen. cDNA was synthesized using the miRCURY LNA RT kit (Qiagen), making sure that equal amounts of miRNA input between samples were used. The level of mature miRNA was assessed using the miRNA LNA primers for miR-155-5p (YP00204308; Qiagen). Other primers used included miR-19b-3p (YP00204450), miR-195-5p (YP00205869), miR-124-3p (YP00206026), miR-185-5p (YP00206037), miR-151a-5p (YP00204007), miR-126-3p (YP00204227), miR-210-3p (YP00204333), and let-7a-5p (YD00610604). PCR reactions were performed in triplicate in 10-µl volumes in 384-well plates. EV content of specific miRNAs varied widely because of both the total amount of EV and the relative abundance of any particular miRNA under different conditions. Thus, when comparing two conditions, changes in EV miRNA are presented as “fold EV miR enrichment.” Because there is no accepted constant endogenous transcript control for EVs, we accomplished this by normalizing miRNA content to the total RNA content of the EV preparation. EV RNA was isolated from cell supernatants as described, and an equal RNA quantity (0.5 µg of total RNA) was used as input for cDNA synthesis. The relative miRNA enrichment after an experimental manipulation was then calculated as fold miRNA enrichment = miRNA quantity_experimental_/miRNA quantity_control_, where miRNA quantity was determined by qRT-PCR using a constant input of total RNA.

### RNA binding assay

We first generated sgTRIM25 HeLa cells by performing CRISPR-mediated knock-out of TRIM25. To this, we transfected cells with px458_2A_GFP_sgRNA_TRIM25_G1 (a gift from Thomas Tuschl, Addgene plasmid # 127119). After 24 h, GFP-positive cells were sorted clonally by FACS into 96-well plates and cultured until colonies were obtained. Clonal cell lines that grew from single colonies were analyzed by western blot for TRIM25 depletion. To perform the RNA binding assay, HeLa or sgTRIM25 HeLa cells were treated with Sendai Virus for 24 h and lysed in RIPA buffer containing protease inhibitor. FMR1 was immunoprecipitated from 3 mg of a total cell lysate in a final volume of 700 µl using 14 µl FMRP Antibody (Cell Signaling Technology, 4317) overnight at 4 °C. 150 µl of M-280 Sheep Anti-Rabbit IgG dynabeads (Fisher, 11203D) were added and incubated with gentle shaking for 4 h at 4 °C. The beads were washed five times using RIPA lysis buffer, resuspended in 50 µl RIPA lysis buffer and frozen in 2 µl aliquots. To perform the RNA binding assay, 0.5 µl corresponding to 15 ng of bead-bound FMR1 was incubated with 1 nM labeled RNA oligo and 1 U/µl SUPERase·In RNase Inhibitor. Oligos were purchased from IDT (Coralville, Iowa) and included miR155-5p-3′FAM (rUrUrArArUrGrCrUrArArUrCrGrUrGrArUrArGrGrGrGrUrU/36-FAM/) and miR155mut-5p-3′FAM (rUrUrGrArUrGrCrUrArArUrCrGrUrGrArUrArGrGrGrGrUrU/36-FAM/). The sample was brought to 50 µl using equilibration buffer (0.01% IGEPAL, 0.01 mg/ml tRNA, 10 mM Tris, pH 8.0, 100 mM NaCl) and incubated for 3 h at room temperature. The samples were washed twice in equilibration buffer and resuspended in 100 µl equilibration buffer supplemented with 62.5 mM Tris, 10 mM glycerol, 5% β-Mercaptoethanol, and 1% SDS. The samples were incubated at 100 °C for 5 min and the 3’FAM tagged RNA was detected in the supernatant using a Fluostar Optima plate reader (BMG Labtech, Durham, NC). Fluorescence measurements were performed at room temperature at excitation wavelength 485 and emission of 520. To calculate % bound oligo, a free solution calibration was performed. The 3′-FAM tagged oligo of known concentrations was diluted in equilibration buffer and the fluorescence intensity was measured with the plate reader as described. The calibration curve was well fitted by the equation nM oligo = [(F − b)/m] where F is the fluorescence intensity reading and m and b are constants. Fluorescence values of the samples were converted to nM bound oligo by extrapolation of the calibration curve.

### miR-155 activity

HMC3 cells were transfected with control or shTrim25 plasmid. The cells were treated with 1 µg/ml LPS (serotype O55:B5, Enzo) for 3 h and then stimulated with 5 mM ATP for an additional 60 min to activate the inflammasome. After 24 h, the conditioned media from these cells was used to treat cells expressing 155_KRAS in pmiRGlo (Addgene plasmid # 78132). This plasmid contains the 3′-UTR of KRAS comprising two miR-155 binding sites. After 2 h of treatment, the cells were collected in RIPA buffer and luciferase activity was measured using Promega’s Dual-Luciferase Reporter Assay System (E1960) on a Promega GloMax. Firefly luciferase corresponding to 155_KRAS was normalized to an independently transcribed Renilla luciferase.

### Statistics

All experimental results represent observations from at least three biological replicates. Unless indicated otherwise, all data are presented as mean ± SEM; the number of replicates is indicated in the legends. Student’s t test was used for statistical analyses. *p* ≤ 0.05 was considered significant.

### Supplementary Information

Supplementary Figure 1.

## Data Availability

The authors declare that data supporting the findings of this study are available within the article and are available from the corresponding author upon request.

## References

[1] Gupta A, Pulliam L (2014). Exosomes as mediators of neuroinflammation. J. Neuroinflamm..

[2] Villarroya-Beltri C, Baixauli F, Gutiérrez-Vázquez C, Sánchez-Madrid F, Mittelbrunn M (2014). Sorting it out: Regulation of exosome loading. Semin. Cancer Biol..

[3] Shields SB, Piper RC (2011). How ubiquitin functions with ESCRTs. Traffic (Copenhagen, Denmark).

[4] Swatek KN, Komander D (2016). Ubiquitin modifications. Cell Res..

[5] Sun T, Liu Z, Yang Q (2020). The role of ubiquitination and deubiquitination in cancer metabolism. Mol. Cancer.

[6] Choudhury NR, Heikel G, Michlewski G (2020). TRIM25 and its emerging RNA-binding roles in antiviral defense. Wiley Interdiscip. Rev. RNA.

[7] Martín-Vicente M, Medrano LM, Resino S, García-Sastre A, Martínez I (2017). TRIM25 in the regulation of the antiviral innate immunity. Front. Immunol..

[8] Gack MU (2007). TRIM25 RING-finger E3 ubiquitin ligase is essential for RIG-I-mediated antiviral activity. Nature.

[9] Choudhury NR (2014). Trim25 is an RNA-specific activator of Lin28a/TuT4-mediated uridylation. Cell Rep..

[10] Zheng X (2017). TRIM25 is required for the antiviral activity of zinc finger antiviral protein. J. Virol..

[11] Gebauer F, Schwarzl T, Valcárcel J, Hentze MW (2021). RNA-binding proteins in human genetic disease. Nat. Rev. Genet..

[12] Hentze MW, Castello A, Schwarzl T, Preiss T (2018). A brave new world of RNA-binding proteins. Nat. Rev. Mol. Cell Biol..

[13] Gibbings DJ, Ciaudo C, Erhardt M, Voinnet O (2009). Multivesicular bodies associate with components of miRNA effector complexes and modulate miRNA activity. Nat. Cell Biol..

[14] Irion U, St Johnston D (2007). bicoid RNA localization requires specific binding of an endosomal sorting complex. Nature.

[15] Wozniak AL (2020). The RNA binding protein FMR1 controls selective exosomal miRNA cargo loading during inflammation. J. Cell Boil..

[16] Jordens I (2001). The Rab7 effector protein RILP controls lysosomal transport by inducing the recruitment of dynein-dynactin motors. Curr. Biol..

[17] Adams A, Weinman SA, Wozniak AL (2018). Caspase-1 regulates cellular trafficking via cleavage of the Rab7 adaptor protein RILP. Biochem. Biophys. Res. Commun..

[18] Takashiba S (1999). Differentiation of monocytes to macrophages primes cells for lipopolysaccharide stimulation via accumulation of cytoplasmic nuclear factor kappaB. Infect. Immun..

[19] Bala S (2012). Circulating microRNAs in exosomes indicate hepatocyte injury and inflammation in alcoholic, drug-induced, and inflammatory liver diseases. Hepatology (Baltimore, Md.).

[20] Eguchi A, Feldstein AE (2018). Extracellular vesicles in non-alcoholic and alcoholic fatty liver diseases. Liver Res..

[21] Zhang J (2015). Exosome and exosomal microRNA: Trafficking, sorting, and function. Genom. Proteom. Bioinform..

[22] Putz U (2008). Nedd4 family-interacting protein 1 (Ndfip1) is required for the exosomal secretion of Nedd4 family proteins. J. Biol. Chem..

[23] Kunadt M (2015). Extracellular vesicle sorting of α-Synuclein is regulated by sumoylation. Acta Neuropathol..

[24] Villarroya-Beltri C (2016). ISGylation controls exosome secretion by promoting lysosomal degradation of MVB proteins. Nat. Commun..

[25] Huang X, Dixit VM (2016). Drugging the undruggables: Exploring the ubiquitin system for drug development. Cell Res..

[26] Mahesh G, Biswas R (2019). MicroRNA-155: A master regulator of inflammation. J. Interf. Cytokine Res..

[27] Wozniak AL (2010). Intracellular proton conductance of the hepatitis C virus p7 protein and its contribution to infectious virus production. PLoS Pathog..

[28] Ascano M (2012). FMRP targets distinct mRNA sequence elements to regulate protein expression. Nature.

[29] Lim KL (2005). Parkin mediates nonclassical, proteasomal-independent ubiquitination of synphilin-1: Implications for Lewy body formation. J. Neurosci..

[30] Eichelser C (2014). Increased serum levels of circulating exosomal microRNA-373 in receptor-negative breast cancer patients. Oncotarget.

[31] Thery C, Amigorena S, Raposo G, Clayton A (2006). Isolation and characterization of exosomes from cell culture supernatants and biological fluids. Curr. Protoc. Cell Boil..

