# Supplementary material for: TRIM25 dictates selective miRNA loading into extracellular vesicles during inflammation
Source: Sci Rep. 2023 Dec 22;13:22952. doi: 10.1038/s41598-023-50336-5 (PMC10746700; doi:10.1038/s41598-023-50336-5)
Supplement: Supplementary file 1 — Supplementary Figure 1. [file 41598_2023_50336_MOESM1_ESM.pdf]

TRIM25 Dictates Selective miRNA Loading into Extracellular Vesicles During Inflammation

Kayla E. King[1,2], Priyanka Ghosh[1,2], and Ann L. Wozniak[*1,2]

[1]Department of Internal Medicine, University of Kansas Medical Center, Kansas City KS, 66160, U.S.A
[2]Liver Center, University of Kansas Medical Center, Kansas City KS, 66160, U.S.A

Corresponding author:
Ann L. Wozniak, Ph.D.
Department of Internal Medicine
University of Kansas Medical Center, Mailstop 1018
Kansas City, KS 66160
Tel: 913-588-4760
email: awozniak@kumc.edu

Supplementary Figure 1. Uncropped Gels

Fig 1A, Left Panel

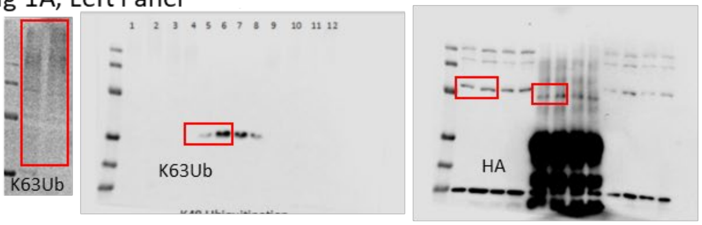

Fig A, Right Panel

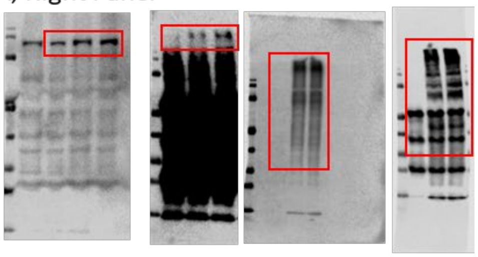

Fig 1B

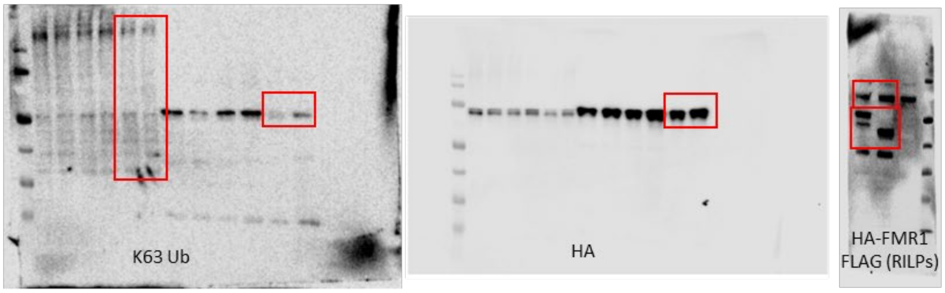

Fig 1E

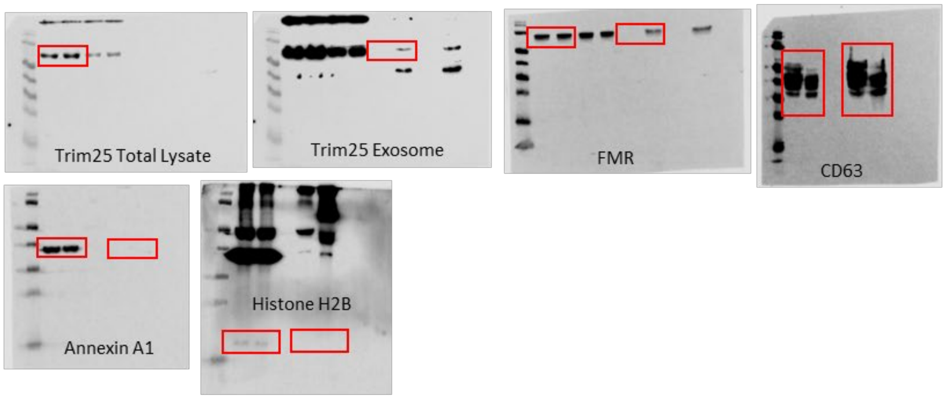

Fig 1F

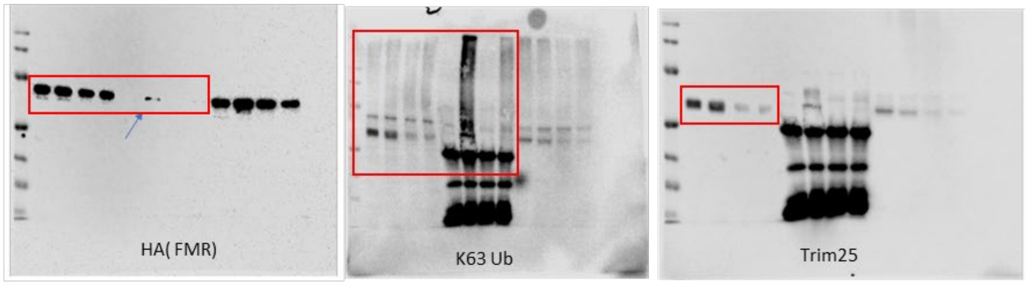

Supplementary Figure 1 cont. Uncropped Gels

Fig 2A

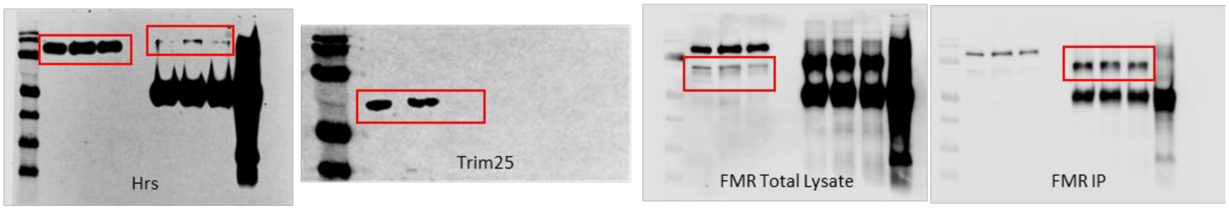

Fig 2c

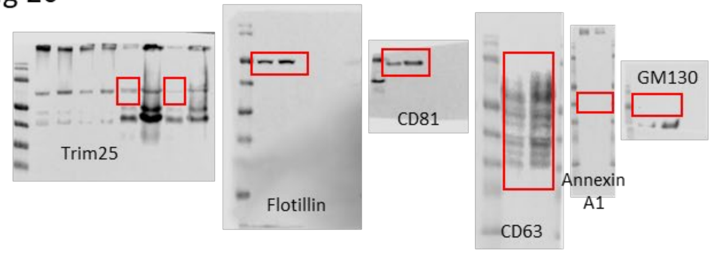

Fig 2D

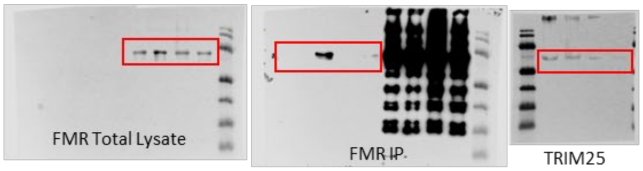

Fig 3D

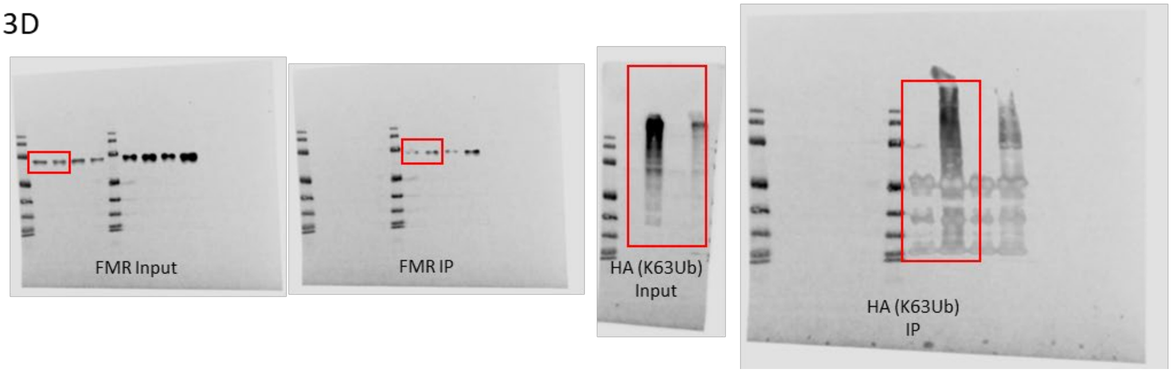

Supplementary Fig. 1. Original blots used in the manuscript. Uncropped and un-processed blots shown in figures 1-3. Routinely membranes were cut prior to incubation with primary antibody to analyze proteins in the upper and lower membranes simultaneously or cut vertically at a marker because multiple samples were run on a single gel. For fig1A, left panel, the K63Ub blot was contrast enhanced and cropped to obtain the input.
